# Lumican silencing alleviates tumor necrosis factor-α-induced nucleus pulposus cell inflammation and senescence by inhibiting apoptosis signal regulating kinase 1/p38 signaling pathway via inactivating Fas ligand expression

**DOI:** 10.1080/21655979.2021.1973781

**Published:** 2021-09-13

**Authors:** Zhenqiang Li, Chengfeng Sun, Maosong Chen, Boding Wang

**Affiliations:** Department of Neurosurgery, Ningbo Medical Center Lihuili Hospital, Ningbo, Zhejiang, China

**Keywords:** Lumican, intervertebral disc degeneration, nucleus pulposus cells, cell senescence, cell cycle

## Abstract

A recent study has reported that lumican (LUM) is expressed at a high level in the nucleus pulposus specimens from herniated lumbar disc, without description of the specific mechanism. This study was designed to investigate the function and mechanism of LUM in intervertebral disc degeneration (IDD). In this study, human nucleus pulposus cells (hNPCs) cells were challenged with tumor necrosis factor (TNF)-α to establish the IDD in vitro model. After LUM silencing, cell viability was detected using CCK-8 kit, and the expression of inflammatory factors was evaluated using RT-qPCR and ELISA. Flow cytometry and β-galactosidase staining were used to determine cell cycle and cell senescence. The expression of cycle and senescence-related proteins was evaluated with western blotting. Then, Fas ligand (FasL) was overexpressed and proteins in apoptosis signal regulating kinase 1 (ASK1)/p38 signaling were tested. Finally, GS-4997, an inhibitor of ASK1, was used to explore the regulatory effects of LUM on ASK1/p38 signaling in TNF-α-induced hNPCs. Results indicated that LUM expression was upregulated in TNF-α-challenged hNPCs. LUM gene interference mitigated TNF-α-induced inflammatory response, cell cycle arrest, and senescence of hNPCs. It was then found that LUM silencing could inhibit ASK1/p38 signaling in TNF-α-treated hNPCs, which was reversed by FasL overexpression. Additionally, ASK1/p38 participated in the mediation by LUM of TNF-α-induced inflammation, cell cycle arrest, and senescence of hNPCs. To conclude, interference with LUM effectively mitigated TNF-α-induced inflammatory response, cell cycle arrest, and cell senescence. Further experiments showed the involvement of ASK1/p38 pathway in LUM-mediated NP cell phenotypes through FasL.

## Introduction

Low back pain is the most typical symptom of musculoskeletal spinal disease and is characterized by pain, stiffness, muscle tension between the costal margin and the subgluteal fold and even pain in the nerve roots of the lower leg just below the knee [1]. Among all the musculoskeletal spinal diseases, intervertebral disc degeneration (IDD) is the chief cause of clinical cases of low back pain [2]. Once the intervertebral disc appears to be degenerating, it will lead to the tearing and ossification of cartilage endplate, spinal canal stenosis, vertebral body deformation, and other problems, ultimately resulting in the loss of morphological stability and function of the intervertebral disc [3–5].

The pathological changes of nucleus pulposus (NP) cells and extracellular matrix are also a generally acknowledged explanation of lumbar degenerative diseases. NP is a gelatinous block just to the right of the center of the disc. With age, NP tissue hardens, and the elastic state and the pressure level change as the content of water in NP decreases, leading to alterations in the biomechanical properties of the disc [6]. The inflammatory microenvironment in human nucleus pulposus cells (hNPCs) is the main culprit of extracellular matrix destruction. Intracellular accumulation of proinflammatory cytokines activates inflammation-related signaling pathways and produces cascade reaction, forming a vicious circle that accelerates NP degeneration [7–10]. In addition, the presence of senescent hNPCs has been demonstrated in degenerative intervertebral discs and is considered to be associated with reactive oxygen-free radical-induced oxidative stress [11,12]. It is thus extrapolated that the progression of IDD could be eased by effective inhibition of NP cell inflammation and senescence.

Lumican (LUM), an important extracellular matrix glycoprotein in human cartilage tissues, has been recently reported to be expressed at a differentially high level in the NP specimens from human herniated lumbar discs [13]. A study in 1999 has corroborated a senescence-related increase in LUM in the lumbar intervertebral discs obtained from patients of varying ages [14]. Another study demonstrated that higher mRNA level for LUM analyzed by cDNA array and RT-PCR in scoliotic discs versus normal discs of identical degeneration score [15]. Additionally, LUM has been shown to be increased in various inflammatory-like conditions and to be able to regulate multiple inflammation-related pathways in different diseases, such as osteoarthritis, keratitis, pancreatitis, and heart failure [16–20]. At present, the exact mechanism of LUM in the development of IDD remains to be elucidated. Therefore, this study intended to find out the role of LUM in the inflammatory response and senescence of hNPCs in IDD and to describe the potential mechanism.

In this study, hNPCs were treated with tumor necrosis factor (TNF)-α to establish the IDD in vitro model. Subsequently, LUM expression was determined, and the effects of LUM silencing on the inflammation, proliferation, senescence, and the underlying mechanisms related to Fas ligand (FasL) and apoptosis signal regulating kinase 1 (ASK1)/p38 signaling were explored.

## Materials and methods

### Cell culture and treatment

hNPCs (Procell, Wuhan, China) were cultured in Dulbecco’s modified Eagle’s medium (DMEM)/F12 medium supplemented with 10% phosphate buffer saline (PBS) and 1% penicillin and streptomycin. TNF-α (Sigma-Aldrich) in 10 ng/mL was chosen to stimulate hNPCs. 10 µM of ASK1 inhibitor (GS-4997) was added to incubate with the cells for suppression of ASK1 and p38 phosphorylation.

### Cell transfection

The transfection of small interfering RNA (siRNA) targeting LUM (si-LUM-1 or si-LUM-2), its negative control (si-NC), FasL overexpressing pcDNA 3.1 (Oe-FasL) and the empty plasmid (Oe-NC) (all synthesized by GenePharma, Shanghai, China), was carried out using Lipofectamine 3000 (Invitrogen) in accordance with the product instructions. After 48 h of transfection, the cells were harvested and the expression aforementioned genes was tested by means of reverse transcription-quantitative PCR (RT-qPCR) and western blotting.

### Enzyme-linked immunosorbent assay (ELISA)

The concentrations of interleukin (IL)-1β, IL-6 and TNF-α in hNPCs of different groups were measured with commercial ELISA kit (Shanghai XiTang Biotechnology, Shanghai, China) according to the manufacturer’s guidelines.

Telomerase activity was analyzed using the double antibody sandwich ABC-ELISA method using the Telomerase ELISA kit (Shanghai XiTang Biotechnology, Shanghai, China). Antihuman telomerase monoclonal antibody was coated on the microplate to combine with the telomerase in the standards and the samples. The biotin antihuman telomerase was added to form the immune complex. After the binding of horseradish peroxidase labeled Streptavidin and biotin, the staining solution followed by sulfuric acid was added to terminate the reaction. OD value at 450 nm was measured with a microplate reader, and the concentration of telomerase was proportional to the OD value.

### Cell counting kit-8 (CCK-8) assay

Cell viability was evaluated with a CCK-8 kit (Shanghai YiSheng Biotechnology Co., Ltd., Shanghai, China). The treated cells were cultured in 96-well plates for 24 h. CCK-8 reagent of 10 μl was then added to each well, and the plate was kept in the incubator for 2 h. The absorbance was measured at 450 nm with a multi-function microplate analyzer.

### Flow cytometry

For cell cycle analyses, trypsin-treated hNPCs in different groups were fixed in 70% ethanol at 4°C for 4 h and stained with 50 μg/mL RNase A and 50 μg/mL PI for 30 min at 37°C. Samples were analyzed with a flow cytometer (Beckman Coulter, Brea, CA, USA).

### β-galactosidase staining

hNPCs were inoculated in 6-well plates. After removal of the culture medium and a wash with PBS, the cells were fixed at room temperature for 20 min and washed with PBS for 3 min/3 times. After addition of 1 ml of β-galactosidase staining solution (Aladdin, Shanghai, China) to each well, the cells were incubated at 37°C. β-galactosidase-positive cells were observed under a microscope the next day.

### RT-qPCR

Total RNA was extracted from each group according to the instructions of the RNA extraction kit (GeneSeed, Guangzhou, China) for reverse transcription. Complementary DNA (cDNA) amplification was performed on a PCR instrument. Then, PCR experiments were performed using SYBR Green PCR Master Mix (Applied Biosystems) on an ABI 7300 thermal-recycler (Applied Biosystems; Thermo Fisher Scientific, Inc.). GAPDH was used as internal controls for normalization. The relative mRNA expression was calculated using the 2^−ΔΔCq^ method [21].

### Western blot analysis

Total protein of each group was extracted by the use of lysis buffer RIPA lysis buffer (Beyotime, Shanghai, China) followed by centrifugation, and the protein concentration was determined by a bicinchoninic acid (BCA) method (Beyotime, Shanghai, China). After thermal denaturation, samples of equal quantity were taken for 10% sodium dodecyl sulfate-polyacrylamide gel electrophoresis (SDS-PAGE), and transferred onto nitrocellulose membranes. Possible nonspecific binding was blocked by 5% nonfat milk and then incubated overnight at 4°C with specific primary antibodies. The secondary antibody was added the next day and incubated at room temperature for 1 h. Finally, the bands were visualized using an Odyssey Infrared Imaging Scanner (LI-COR Biosciences). Band densities of target proteins were normalized to that of GAPDH and quantified using Image J software.

### Statistical analysis

GraphPad Prism 8.0 was used for statistical analysis. The measurement data were expressed in the form of the mean ± deviation and were repeated 3 times for each sample. The comparison employed one-way one-away analysis of variance (ANOVA) followed by Tukey’s post hoc test for multiple groups and student’s t-test for two groups. P < 0.05 was the test standard.

## Results

### The level of LUM was increased in TNF-α-challenged hNPCs and LUM deletion reduced TNF-α-induced inflammatory response in hNPCs

**Figure 1.**
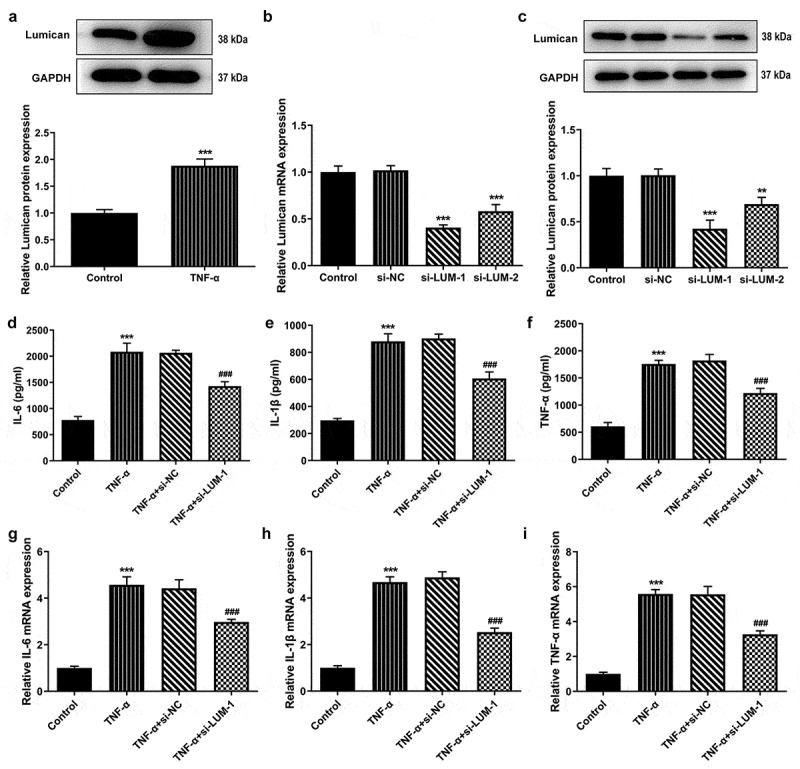
LUM expression was notably decreased in TNF-α-induced hNPCs and LUM deletion reduced TNF-α-induced inflammatory response in hNPCs. (a) Detection of LUM protein expression by means of western blot in the control group and in hNPCs stimulated with TNF-α. N = 3. ***P < 0.001 vs. control. (b–c) Detection of the interference efficacy of si-LUM1/2 by means of RT-qPCR and western blot analysis in hNPCs. N = 3. **P < 0.01, ***P < 0.001 vs. si-NC. (d–f) The concentrations of pro-inflammatory cytokine levels in hNPCs stimulated with TNF-α or transfected with si-NC/si-LUM-1was examined with ELISA kits. (g–i) RT-qPCR was used to evaluate the mRNA expression of pro-inflammatory cytokine in hNPCs stimulated with TNF-α or transfected with si-NC/si-LUM-1. N = 3. ***P < 0.001 vs. control; ^###^P < 0.001 vs. TNF-α+ si-NC

As an important extracellular matrix glycoprotein in human cartilage tissues, LUM has been demonstrated to be highly expressed in the NP specimens from human herniated lumbar discs [13]. However, the role of LUM in the progression of IDD remains to be elucidated. Firstly, LUM expression was tested using western blot analysis in hNPCs after stimulation with TNF-α. An increase in the level of LUM in hNPCs was observed in the TNF-α group, compared to the control group (Figure 1(a)). This implies that there exists a possible upregulation of LUM expression in IDD. Then, LUM was silenced by transfection with si-LUM-1 and 2. The results of RT-qPCR and western blotting first proved the interference efficacy of si-LUM-1 and 2 and showed that si-LUM-1 had a better knockdown effect than si-LUM-2 (Figure 1). Si-LUM-1 was thus selected for the subsequent experiments. Moreover, as displayed in Figure 1(d–i), the concentrations and mRNA expression levels of pro-inflammatory cytokines IL-6, IL-1β and TNF-α were notably upregulated after TNF-α stimulation but were downregulated by LUM interference in TNF-α-stimulated hNPCs. Therefore, it can be inferred that LUM knockdown could reduce TNF-α-induced inflammatory response in hNPCs.

### LUM deletion alleviated TNF-α-induced cell cycle arrest and cellular senescence of hNPCs

**Figure 2.**
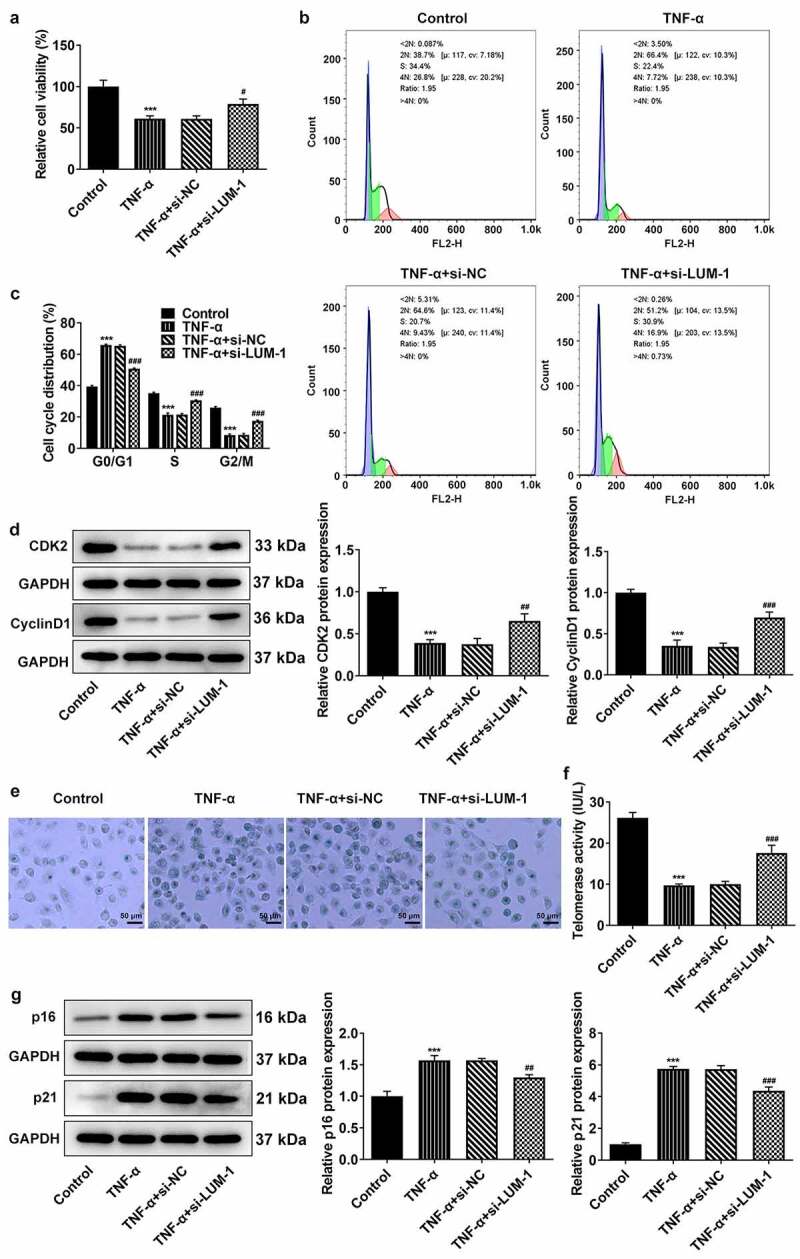
**LUM deletion alleviated TNF-α-induced cell cycle arrest and cellular senescence of hNPCs**. Analysis of cell viability (a), cell cycle distribution (b-c) and the expression of cell cycle markers (d) by use of CCK-8, flow cytometry and western blot in hNPCs stimulated with TNF-α or transfected with si-NC/si-LUM-1. Analysis of senescent cells (e), telomerase activity (f) and the expression of senescence markers (g) by means of β-galactosidase staining, telomerase ELISA and western blot in hNPCs stimulated with TNF-α or transfected with si-NC/si-LUM-1. N = 3. ***P < 0.001 vs. control; ^#^P < 0.05, ^##^P < 0.01, ^###^P < 0.001 vs. TNF-α+ si-NC

Age-related cellular senescence is most likely the cause of NP cell apoptosis in IDD [22]. The state of cell cycle can well reflect the process of cellular senescence [23]. Analysis of cell viability by the use of CCK-8 found that the TNF-α induction led to a significant decrease in cell viability when compared to the control group. By contrast, interference with LUM increased TNF-α-impaired viability of hNPCs (Figure 2(a)). Furthermore, stimulation with TNF-α-induced cell cycle arrest of hNPCs, as evidenced by increased cell numbers in the G0/G1 phase and the noticeable decline in cell numbers in the S and G2/M phases (Figure 2). However, LUM interference reversed these changes caused by TNF-α to a significant degree. Additionally, cell cycle drivers cyclin-dependent kinase 2 (CDK2) and Cyclin D1 were also found to be expressed at a low level in hNPCs challenged with TNF-α compared to the control, whereas si-LUM-1 elevated their expression levels (Figure 2(d)). Moreover, during the observation of cellular senescence, it was found through β-galactosidase staining that the number of senescent hNPCs increased after stimulation with TNF-α and that the number was considerably less in the si-LUM-1 group than in the control group (Figure 2(e)). Besides, the telomerase activity in hNPCs was also suppressed by TNF-α but largely restored by LUM knockdown in TNF-α-stimulated hNPCs (Figure 2(f)). Furthermore, the results of western blot detected an increase in the levels of senescence markers p16 and p21 after TNF-α stimulation and a decrease in their levels in TNF-α-stimulated hNPCs transfected with si-LUM-1 (Figure 2(g)). These results indicate that downregulation of LUM expression alleviated TNF-α-induced cell cycle arrest and cellular senescence of hNPCs.

### LUM silencing downregulated ASK1/p38 signaling pathway through FasL in TNF-α-induced hNPCs

**Figure 3.**
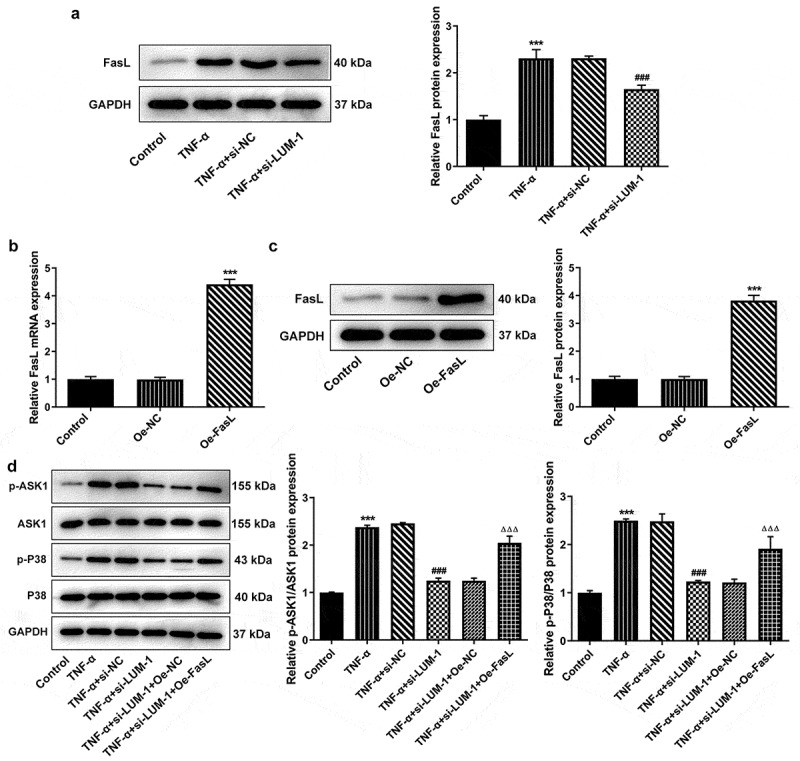
LUM silencing downregulated ASK1/P38 signaling pathway through FasL in TNF-α-induced hNPCs. (a) Detection of FasL expression level by western blot in hNPCs stimulated with TNF-α or transfected with si-NC/si-LUM-1. N = 3. ***P < 0.001 vs. control; ^###^P < 0.001 vs. TNF-α+ si-NC. (b–c) Detection of the overexpression efficacy of Oe-FasL by means of RT-qPCR and western blot assay. N = 3. ***P < 0.001 vs. Oe-NC. (d) The expression of phosphorylated ASK1 and p38 detected by western blot in TNF-α-stimulated hNPCs transfected with si-NC/si-LUM-1 or co-transfected with si-LUM-1 and Oe-NC/FasL. N = 3. ***P < 0.001 vs. control; ^###^P < 0.001 vs. TNF-α+ si-NC; ^ΔΔΔ^P<0.001 vs. TNF-α+ si-LUM-1+ Oe-NC

It has been reported that LUM can be co-immunoprecipitated with FasL to induce fibroblast apoptosis and inflammatory response [24]. Noteworthy, exogenous FasL could diminish the protective effect of TGF-β1 on TNF-α-challenged hNPCs [25]. Additionally, the bioinformatics analysis employing KEGG database (https://www.kegg.jp/) found that FasL might regulate the expression of downstream ASK1 and p38 together identified as a key pathway of cell apoptosis and senescence [26]. Therefore, the expression of FasL and its regulatory effects on ASK1/p38 signaling were explored in this study. FasL expression was found to be high in the TNF-α group but lowered by LUM interference in TNF-α-challenged hNPCs (Figure 3(a)). To perform the gain-of-function experiments on FasL, FasL expression was upregulated by use of its overexpression plasmid (Figure 3). Subsequently, TNF-α-upregulated expression levels of phosphorylated ASK1 and p38 were shown to be decreased by the knockdown of LUM but reversed after co-transfection of si-LUM-1 and Oe-FasL (Figure 3(d)). These results suggest that the regulation of ASK1/p38 signaling pathway by LUM could be mediated by FasL in hNPCs.

### LUM silencing alleviated TNF-α-induced inflammatory response, cell cycle arrest and cellular senescence of hNPCs by inhibiting ASK1/p38 signaling pathway via inactivating FasL expression

**Figure 4.**
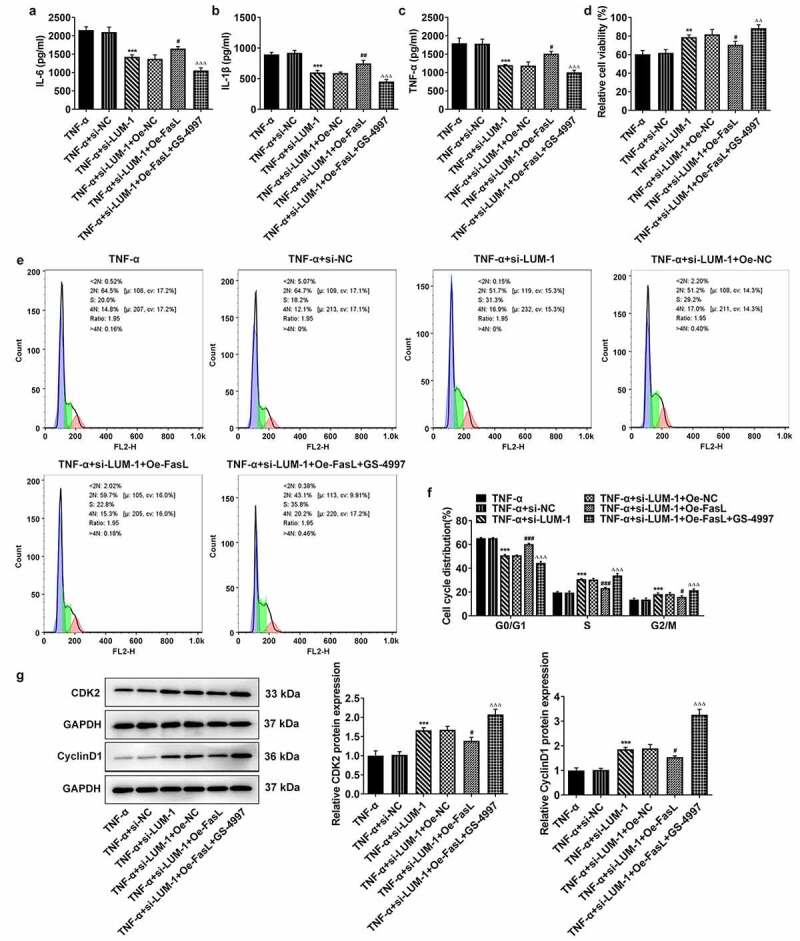
LUM silencing alleviated TNF-α-induced inflammatory response and cell cycle arrest of hNPCs by inhibiting ASK1/p38 signaling pathway via inactivating FasL expression. (a–c) The contents of pro-inflammatory cytokine was detected by ELISA in TNF-α-stimulated hNPCs transfected with si-NC/si-LUM-1 or co-transfected with si-LUM-1 and Oe-NC/FasL. Analysis of cell viability (d), cell cycle distribution (e–f) and the expression of cell cycle markers (g) by use of CCK-8, flow cytometry and western blot in TNF-α-stimulated hNPCs transfected with si-NC/si-LUM-1 or co-transfected with si-LUM-1 and Oe-NC/FasL. N = 3. **P < 0.01, ***P < 0.001 vs. TNF-α+ si-NC; ^#^P < 0.05, ^##^P < 0.01, ^###^P < 0.001 vs. TNF-α+ si-LUM-1+ Oe-NC; ^ΔΔΔ^P<0.001 vs. TNF-α+ si-LUM-1+ Oe-FasL

**Figure 5.**
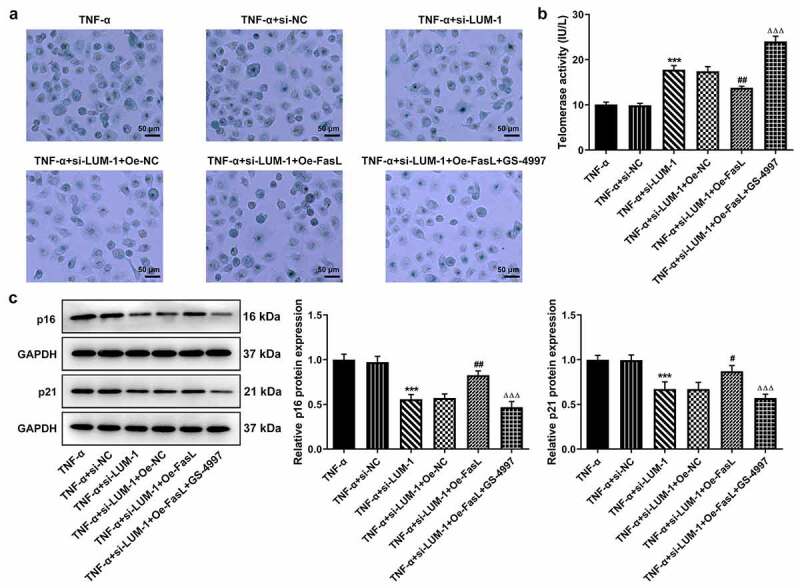
LUM silencing attenuated TNF-α-induced cellular senescence of hNPCs by inhibiting ASK1/p38 signaling pathway via inactivating FasL expression. Analysis of senescent cells (a), telomerase activity (b) and the expression of senescence markers (c) by means of β-galactosidase staining, telomerase ELISA and western blot in TNF-α-stimulated hNPCs transfected with si-NC/si-LUM-1 or co-transfected with si-LUM-1 and Oe-NC/FasL. N = 3. ***P < 0.001 vs. TNF-α+ si-NC; ^#^P < 0.05, ^##^P < 0.01 vs. TNF-α+ si-LUM-1+ Oe-NC; ^ΔΔΔ^P<0.001 vs. TNF-α+ si-LUM-1+ Oe-FasL

To further clarify the direct relationship between LUM and the ASK1/p38 pathway in IDD, hNPCs were treated with GS-4997, a highly selective and effective inhibitor of ASK1, to inhibit the phosphorylation of ASK1 and p38. It was found that while FasL overexpression reversed LUM depletion-downregulated pro-inflammatory cytokine levels, additional GS-4997 decreased the expression of pro-inflammatory cytokines in hNPCs (Figure 4(a–c)). Moreover, in TNF-α-stimulated hNPCs transfected with si-LUM-1, FasL overexpression led to decreased cell viability in contrast with Oe-NC, and an increase in cell viability was observed in the presence of GS-4997 (Figure 4(d)). Co-transfection of si-LUM-1 and Oe-FasL also facilitated cell cycle arrest and suppressed the expression of CDK2 and Cyclin D1 in TNF-α-stimulated hNPCs in comparison with Oe-NC, but such changes were reversed by GS-4997 (Figure 4(e–g)). Additionally, it was observed by means of β-galactosidase staining that, in TNF-α-stimulated hNPCs co-transfected with si-LUM-1 and Oe-FasL, the cellular senescence level increased in comparison with Oe-NC but decreased in the presence of GS-4997 (Figure 5(a)). Furthermore, co-transfection of si-LUM-1 and Oe-FasL led to inhibited telomerase activity in TNF-α-stimulated hNPCs, compared to Oe-NC, whereas GS-4779 reversed this change of trend to a certain degree (Figure 5(b)). By contrast with Oe-NC, the levels of senescence markers p16 and p53 were upregulated by co-transfection of si-LUM-1 and Oe-FasL (Figure 5(c)). However, this effect of FasL overexpression was largely weakened by GS-4997. The results collectively suggest that LUM might participate in TNF-α-induced inflammatory response, cell cycle arrest and cellular senescence of hNPCs through ASK1/p38 signaling pathway via regulating FasL expression.

## Discussion

IDD is an age-related chronic disease in that the flexibility and stability of the intervertebral disc deteriorate as one ages, and the state of hNPCs plays a critical role in this process [27]. IDD progresses slowly and features excessive secretion of pro-inflammatory cytokines, collagen loss, and alterations of NP cell phenotypes [3,28,29]. The severely degenerated disc is in a state of intensive oxidative stress resulting in NP cell apoptosis [30]. Age-related cellular senescence is most likely the cause of NP cell apoptosis in IDD [22]. Studies have found that tumor necrosis factor alpha (TNF-α) is capable of accelerating the senescent process of cells [31]. TNF-α is a known trigger for the inflammatory response in human diseases and is also partly responsible for the nerve pain/low back pain as a manifestation of inflammation in IDD [32,33]. Thus, the present study chose to stimulate hNPCs with TNF-α to establish the in vitro IDD model. As expected, hNPCs stimulated with TNF-α showed a rise in the levels of pro-inflammatory cytokines and cellular senescence, a decline in cell viability and a halt to the cell cycle at the G1 phase. Therefore, TNF-α induced the inflammatory response, cell cycle arrest, and cellular senescence of hNPCs.

LUM has been recently reported to be expressed at a differentially high level in the NP specimens from human herniated lumbar discs [13]. In osteoarthritis, overexpressed LUM has a proven association with the cartilage degradation and macrophage polarization [16]. Furthermore, the secretion of LUM in fibroblasts can be promoted by TNF-α to facilitate the differentiation of fibrocytes [34]. In the present study, an increased expression level of LUM was confirmed in TNF-α-challenged hNPCs. Further knockdown of LUM level decreased the expression of pro-inflammatory molecules, increased the viability level, changed the distribution of cell numbers in different cell cycle phases, and alleviated cellular senescence in TNF-α-challenged hNPCs. It can be inferred from these results that LUM is implicated in NP cell inflammation and senescence in IDD.

Earlier studies have demonstrated that increased proliferation and decreased apoptosis of corneal fibroblasts in LUM-absent mice, and that LUM could be co-immunoprecipitated with FasL to induce fibroblast apoptosis and inflammatory response [24,35]. Noteworthy, exogenous FasL could diminish the protective effect of TGF-β1 on TNF-α-challenged hNPCs [25]. Our results showed that the level of FasL was much higher in the TNF-α group than in the control group, whereas interference with LUM brought down the level of FasL in TNF-α-challenged hNPCs. Moreover, bioinformatics analysis employing KEGG database (https://www.kegg.jp/) found that FasL might regulate the expression of downstream ASK1 and p38 together identified as a key pathway of cell apoptosis and senescence [26]. In the present study, LUM knockdown inhibited TNF-α-induced phosphorylation of ASK1 and p38, whereas FasL overexpression increased the levels of phosphorylated ASK1 and p38 in TNF-α-stimulated hNPCs transfected with si-LUM-1. These results illustrated FasL-mediated LUM regulation of ASK1/p38 signaling pathway. Moreover, TNF-α-stimulated hNPCs co-transfected with si-LUM-1 and Oe-FasL also exhibited higher inflammation level, decreased viability, halted cell cycle at G0/G1, and accelerated cellular senescence. However, the addition of ASK1 inhibitor GS-4997, which functions to prevent the phosphorylation of ASK1 and p38 [36], could effectively reverse these changes. These results imply that LUM participates in TNF-α-induced inflammatory response, cell cycle arrest, and cellular senescence of hNPCs most likely through the ASK1/p38 signaling pathway.

## Conclusion

According to the above experimental results, interfering with LUM can effectively reduce inflammatory response and mitigate cell cycle arrest in TNF-α-stimulated hNPCs. In addition, the participation of LUM in TNF-α-induced hNPCs inflammation and senescence may be explained by FasL-regulated downstream ASK1/p38 signaling pathway. The present study for the first time elucidated the potential role and action mechanism of LUM in IDD and shall serve as an introductory work for future in vivo and in vitro research on the therapeutic value of LUM and FasL for IDD.

## Data Availability

The datasets used and analyzed are available from the corresponding author on reasonable request.
